# The role of histology on endometrial cancer survival disparities in diverse Florida

**DOI:** 10.1371/journal.pone.0236402

**Published:** 2020-07-23

**Authors:** Ariana L. Johnson, Heidy N. Medina, Matthew P. Schlumbrecht, Isildinha Reis, Erin N. Kobetz, Paulo S. Pinheiro

**Affiliations:** 1 Department of Public Health Sciences, University of Miami Miller School of Medicine, Miami, Florida, United States of America; 2 Sylvester Comprehensive Cancer Center, Department of Public Health Sciences, University of Miami Miller School of Medicine, Miami, Florida, United States of America; QIMR Berghofer Medical Research Institute, AUSTRALIA

## Abstract

**Background:**

Endometrial cancer (EC) mortality is particularly high among non-Hispanic Blacks and is twice that of non-Hispanic Whites. However, comparisons of EC survival outcomes by race/ethnicity are often confounded by histology and grade. Here, we analyze EC survival disparities in multiracial Florida with a focus on EC types (1 and 2) and subtypes, defined according to histology and grade.

**Methods:**

All 27,809 cases of EC diagnosed during 2005–2016 were obtained from the Florida Cancer Registry. Age-standardized, 5-year cause-specific survival by race/ethnicity and histological type were calculated. Fine and Gray competing risk regression was used to estimate sub-distribution hazard ratios (sHRs) for associations between risk of death due to EC and potential predictive factors such as histology/grade, age, stage at diagnosis, and insurance.

**Results:**

Type 2 EC accounted for only 38.7% of all incident EC-cases but 74.6% of all EC-deaths. Blacks were disproportionately affected by type 2 EC (57.6%) compared to Whites, Hispanics, and Asians (35.6%, 37.7%, and 43.0%, respectively). Age-adjusted 5-year survival for types 1 and 2 were 85.3% and 51.6%, respectively; however, there was wide variation within type 2 subtypes, ranging from 60.2% for mixed cell EC to as low as 30.1% for carcinosarcoma. In the multivariable model, Blacks with type 2 EC had a 23% higher risk of death due to EC (sHR: 1.23, 95%CI: 1.12–1.36) compared to Whites.

**Conclusions:**

Population-based analyses should consider the histological heterogeneity of EC because the less common type 2 EC drives racial/ethnic survival disparities in EC. Black women have a higher proportion of more aggressive histological types and an overall higher risk of death due to EC than Whites. To the extent that some of these histological types may be considered different diseases and require specific treatment approaches, further research on etiology and prognosis for detailed type 2 EC subtypes is warranted.

## Introduction

Endometrial cancer (EC) is the fourth most commonly diagnosed cancer among women and the most common malignancy of the female reproductive system in the United States, with nearly 62,000 new cases estimated for 2019 [1, 2]. Since 1999, EC incidence rates have been rising in the United States, with larger increases observed among Hispanics (2.9% annually), Blacks (2.4%), and Asians (2.2%), compared to a 1.1% annual increase in Whites [2]. Mortality rates for EC have also increased across all racial/ethnic groups in the US [2], which is in contrast to reports from other “Western countries” in Europe and Oceania [3, 4]. Moreover, during the same time, there has been no substantial improvement in survival for EC [5, 6], with a stagnating 5-year survival of approximately 75% [7, 8].

EC has been divided into two typologies based on differences in histology and subsequent clinical outcomes [9, 10]. The majority of EC cases are type 1 (i.e. low-grade endometrioid type) which are associated with high levels of circulating estrogens, age, obesity, nulliparity, and unopposed estrogen therapy [11–13]. Type 1 ECs are typically correlated with a relatively favorable prognosis with an approximate five-year survival of 85%. In contrast, the less common, type 2 EC encompasses several histological subtypes including serous, mixed cell, clear cell, and carcinosarcoma (formerly called malignant mixed Mullerian tumors) [10, 14] as well as high-grade endometrioid [15, 16]. Type 2 EC has been recognized as clinically more aggressive and heterogeneous [10, 14–16]. Unlike type 1 EC, the association between type 2 and obesity is unclear, and risk factors for type 2 specific histological subtypes have not been comprehensively studied, partly due to their rarity [14]. While this group has been historically referenced as estrogen-independent, a recent meta-analysis [10] demonstrated uncertainty regarding the pathophysiology of type 2 EC since there were no clear hormonal mechanisms differentiating it from those described for type 1 EC [10].

Currently, EC constitutes one of the major cancer disparities in the U.S., with non-Hispanic Black women experiencing an 80% higher mortality rate compared to non-Hispanic White women [17], despite similar incidence rates. Relative to White women, Black women are consistently diagnosed with later stage, higher-grade, and more aggressive histologic subtypes, particularly those of type 2 EC. These differences in stage, histologic subtype, and grade may partially explain the survival disadvantage observed among Black women in relation to White women, which in turn contributes to the higher mortality rate among Blacks [18–20].

In Florida, racial/ethnic EC survival disparities between Whites, Blacks, Hispanics, and Asians have not been addressed in the context of the heterogeneity by histology, grade, or stage at diagnosis. Florida is unique in its demographic composition with Hispanics and Blacks making up approximately 25% and 17% of the population, respectively, with large contingents of Caribbean populations in both groups: Cuban and Puerto Rican among Hispanics, and Afro-Caribbeans among Blacks [21]. All of these subgroups have similar or higher EC mortality rates compared to Whites [22–24], suggesting possible incidence and/or survival disparities that may be unique to Florida.

Using the state’s population-based cancer registry data, the aim of this study was twofold: to assess the role of histology according to type 1 and 2 EC (and its subtypes) in EC survival, and to assess the impact of the distribution of these characteristics on racial-ethnic survival disparities between Whites, Blacks, Hispanics, and Asians in diverse Florida.

## Material and methods

*This is the result of a secondary data analysis with deidentified data. The data was therefore fully anonymized and informed consent was waived. The study is covered under Florida Department of Health IRB #2018–053, PI: PS Pinheiro*.

Data for women diagnosed with a first primary EC from 2005 to 2016 were obtained from the statewide cancer registry, the Florida Cancer Data System (FCDS). FCDS is the legislatively mandated, population-based central cancer registry for Florida. Cases in FCDS are abstracted from patient medical records in hospitals, free-standing ambulatory surgical facilities, radiation therapy facilities, private physicians, and death certificates. The estimated level of completeness is greater than 95%, as determined by external quality control audits. In addition, FCDS has met or exceeded the North American Association of Central Cancer Registries (NAACCR) standards of quality, timeliness, and completeness for every year since 1995 [25]. Cases of primary site codes C54.X and C55.9 and morphology codes 8050–8951 per the International Classification of Diseases for Oncology, third edition (ICD-O-3), were included in this analysis [26].

Variables obtained from FCDS included socio-demographic characteristics, such as sex, age, race/ethnicity, and marital status; as well as tumor characteristics such as stage at diagnosis, histology, and grade. Race/ethnicity was based on self-identification and was present in nearly all (more than 98%) of the health records. It was categorized into four mutually exclusive groups: non-Hispanic White, non-Hispanic Black, and non-Hispanic Asian & Pacific Islander, referred to in this report as Whites, Blacks, Asians for simplicity, and Hispanics, who can be of any race. Cases were not included when the race of the individual was not defined by one of these four groups. Insurance information was classified into five categories: private insurance, Medicare and Specials, Medicaid, no insurance, and unknown. Socio-economic status (SES) was derived from census tract data and categorized into a poverty indicator (following the Krieger scale). Five categories were used: less than 5%, between 5 and 10%, between 10% and 20%, and over 20% poverty amongst the population living in that defined census tract. Surveillance, Epidemiology and End Results (SEER) staging categories (localized, regional, or distant) and tumor histologic subtypes [clear cell (8310), endometrioid (8050, 8140, 8143, 8210–8211, 8260–8263, 8340, 8380–8384, 8560, 8570), mixed cell (8255, 8323), malignant Mullerian mixed tumors (MMMT) and carcinosarcomas (8950–8951, 8980–8981), and serous (8441,8460–8461)] as described in Cote *et al*. [20, 27] were analyzed. Due to the overlap in clinical behavior, prognosis, and etiologic factors shown in previous studies [12, 14, 28], grade 3 and 4 (high-grade) endometrioid cancers were included in type 2 EC [9, 15, 16], while low-grade endometrioid types were included in type 1. In addition to high-grade endometrioid, type 2 included clear cell, mixed high-grade carcinoma, carcinosarcoma, and serous EC [20]. As in similar studies [20], SEER Summary Stage is a collapsed format for staging resulting from FIGO staging categories as follows: localized SEER, FIGO IA, IB, IC, and FIGO stage I not further specified; regional SEER, FIGO stage IIA, IIB, or FIGO stage II, not otherwise specified, FIGO stage IIIA, IIIB, and IIIC; distant SEER, FIGO stage IVA, IVB; and unknown SEER [20, 29]. For follow-up of cancer patients' vital status, FCDS routinely links their cancer cases with death records from both the Florida Department of Health Vital Statistics and the National Death Index. Causes of death were categorized as those due to EC or those due to other causes according to the SEER definition for EC cause of death [30]. Deaths by a cause other than EC were censored at the time of death in the calculation of population-based, five-year cause-specific survival and were treated as a competing risk in a Fine-and-Gray multivariable model. Patients diagnosed at autopsy only or by death certificate were excluded, as were cases with a negative or missing survival period.

Population-based, five-year cause-specific survival was calculated using the lifetable method for the entire population by histology type and race/ethnicity, while adjusting for age according to the International Cancer Survival Standard 5 age-groups: 15–44, 45–54, 55–64, 65–74, and 75+ [31]. Unlike SEER, FCDS does not collect dates of last alive contact. Thus, based on the presumed alive assumption [32], cases that are not found to be deceased on successive annual mortality linkages are censored as alive on the last date covered, in this case, December 31, 2016. Cause-specific survival time was measured in months from the date of diagnosis until the date of death from EC, or December 31, 2016, whichever occurred first.

Finally, Gray's test was used to compare the cumulative incidence curves of EC-specific mortality by race/ethnicity and other categorical variables of interest. The Fine and Gray sub-distribution hazard regression modeling approach [33–35] was used to assess potential predictors of EC-specific mortality in both univariable and multivariable models including age, race/ethnicity, insurance status, census tract poverty, histology, stage, and year of diagnosis. The type-I error was set at 5%. Analyses were performed using SAS 9.4 (SAS Institute Inc., Cary, NC). This study is in compliance with the Florida Department of Health Institutional Review Board.

## Results

A total of 27,089 cases of EC as a first cancer, with a median age at diagnosis of 64 years, were identified in Florida from 2005–2016. The majority were White (69.4%), 51.4% were married, and 47.3% had private insurance (Table 1). By histological type, 61.3% were of type 1 and 39.7% were of type 2 EC. The highest proportion of type 1 was observed among Whites (64.4%). Conversely, the highest proportion of type 2 was seen among Blacks (57.6%), followed by Asians (43.0%), Hispanics (37.7%), and Whites (35.6%). By subtype, Blacks had the highest proportions of carcinosarcoma (12.0%) and serous (12.5%) ECs in comparison to all other racial/ethnic groups. Blacks also had the highest proportion of cases diagnosed at distant stage (12.8%) and were more likely to live in high-poverty areas (48.8%). In regard to insurance, Hispanics had the highest proportion of Medicaid beneficiaries or women without insurance (16.0% and 8.7%, respectively).

**Table 1:** Population characteristics and clinical features of endometrial cancers by race/ethnicity. Florida 2005–2016.

		All-combined		Whites		Blacks		Hispanics		Asians	
		n	%	n	%	n	%	n	%	n	%
**Total**		27,089	100.0	18,806	69.4	3,204	11.8	4,716	17.4	363	1.3
**Age**											
15–44		1,578	5.8	830	4.4	218	6.8	490	10.4	40	11.0
45–54		3,840	14.2	2,560	13.6	371	11.6	827	17.5	82	22.6
55–64		9,007	33.2	6,285	33.4	1,164	36.3	1,440	30.5	118	32.5
65–74		8,042	29.7	5,673	30.2	992	31.0	1,279	27.1	98	27.0
75+		4,622	17.1	3,458	18.4	459	14.3	680	14.4	25	6.9
**Marital Status**											
Single		5,046	18.6	3,047	16.2	987	30.8	955	20.3	57	15.7
Married		13,927	51.4	10,259	54.6	1,107	34.6	2,328	49.4	233	64.2
Separated		272	1.0	125	0.7	65	2.0	82	1.7	-	-
Divorced		2,929	10.8	2,001	10.6	385	12.0	521	11.0	22	6.1
Widowed		4,286	15.8	2,981	15.9	573	17.9	690	14.6	42	11.6
Unmarried or domestic partner		34	0.1	27	0.1	3	0.1	4	0.1	-	-
Unknown		595	2.2	366	1.9	84	2.6	136	2.9	9	2.5
**Insurance**											
Private		12,800	47.3	9,321	49.6	1,272	39.7	2,022	42.9	185	51.0
Medicare and Specials		8,974	33.1	6,611	35.2	1,040	32.5	1,229	26.1	94	25.9
Medicaid		2,159	8.0	913	4.9	455	14.2	753	16.0	38	10.5
No Insurance		1,399	5.2	710	3.8	252	7.9	412	8.7	25	6.9
Unknown		1,757	6.5	1,251	6.7	185	5.8	300	6.4	21	5.8
**Census Tract Poverty**											
0%—<5% poverty		3,916	14.5	3,070	16.3	247	7.7	524	11.1	75	20.7
5%—<10% poverty		7,146	26.4	5,707	30.3	379	11.8	949	20.1	111	30.6
10%—<20% poverty		9,621	35.5	6,755	35.9	970	30.3	1,782	37.8	114	31.4
20% - 100% poverty		6,058	22.4	3,014	16.0	1,564	48.8	1,419	30.1	61	16.8
Unknown or not applicable		348	1.2	260	1.3	44	1.4	42	0.9	2	0.5
**SEER Stage**											
Localized		17,986	66.4	12,852	68.3	1,766	55.1	3,146	66.7	222	61.2
Regional		6,184	22.8	4,167	22.2	867	27.1	1,057	22.4	93	25.6
Distant		1,888	7.0	1,140	6.1	410	12.8	304	6.4	34	9.4
Unknown		1,031	3.8	647	3.4	161	5.0	209	4.4	14	3.9
**Histology**											
Type 1: Low-Grade Endometrioid		16,619	61.3	12,113	64.4	1,359	42.4	2,940	62.3	207	57.0
Type 2: All-combined		10,470	38.7	6,693	35.6	1,845	57.6	1,776	37.7	156	43.0
	High-Grade Endometrioid	5,954	22.0	4,000	21.3	803	25.1	1,077	22.8	74	20.4
	Clear Cell	425	1.6	258	1.4	92	2.9	67	1.4	8	2.2
	Carcinosarcoma	1,372	5.1	758	4.0	384	12.0	209	4.4	21	5.8
	Mixed High Grade	1,082	4.0	743	4.0	164	5.1	156	3.3	19	5.2
	Serous	1,637	6.0	934	5.0	402	12.5	267	5.7	34	9.4

There were 6,069 deaths recorded among all EC cases: 4,100 were due to EC and 1,969 were due to other causes. For the more common type 1 EC (61.3% of all cases), the majority of deaths were due to causes other than EC (n = 1,204) while 1,043 died of EC. Conversely, for type 2, there were 3,057 deaths due to EC versus 657 deaths by other causes. Thus, overall, while type 2 EC accounted for only 38.7% of all new cases, it accounted for 74.6% of all deaths by EC (Table 2). Moreover, a greater proportion of those diagnosed with type 2 (29.2%) experienced death from EC in comparison to only 6.3% of those with type 1. Those with type 2 had a higher proportion of cases diagnosed at the regional (31.4% vs 17.4%, chi-square *p* <0.001) and distant stage (15.1% vs 1.8%, *p*<0.001) compared to patients with type 1, respectively. Conversely, those with type 1 were more often diagnosed at localized stage (78.2%) in comparison to those with type 2 (47.8%). A larger proportion of cases for high-grade endometrioid, clear cell, and mixed high-grade subtypes, were diagnosed at localized stage (Table 2); however, for carcinosarcoma and serous EC, there was a greater proportion of cases diagnosed at regional and distant stages, 35.9% and 24.2%, respectively for carcinosarcoma and 37.4% and 23.0% for serous EC.

**Table 2:** Distribution (N and %) of stage at diagnosis and vital status/cause of death by histological type and subtype. Florida 2005–2016.

	All-Combined	Type 1 EC	Type 2 EC					
		Low-Grade Endometrioid	High-Grade Endometrioid	Clear Cell	Mixed High-Grade	Carcino-sarcoma	Serous	Total
**Total Cases**	27,089	16,619	5,954	425	1,372	1,082	1,637	10,470
**Stage**								
Localized	18,214	13,158	3,214	204	572	473	593	5,056
	66.4%	78.2%	53.4%	47.7%	52.5%	33.9%	35.9%	47.8%
Regional	6,253	2,928	1,675	151	380	501	618	3325
	22.8%	17.4%	27.8%	35.3%	34.9%	35.9%	37.4%	31.4%
Distant	1,905	304	715	54	114	338	380	1,601
	6.9%	1.8%	11.9%	12.6%	10.5%	24.2%	23.0%	15.1%
Unknown	1047	445	415	19	24	84	60	602
	3.8%	2.6%	6.9%	4.4%	2.2%	6.0%	3.6%	5.7%
**Vital status**								
**Dead**	6,069	2,247	1,881	155	783	299	704	3,822
	22.4%	13.5%	31.6%	36.5%	57.1%	27.6%	43.0%	36.5%
From EC	4,100	1,043	1,412	121	235	692	597	3,057
	15.1%	6.3%	23.7%	28.5%	21.7%	50.4%	36.5%	29.2%
Other Causes	1,969	1,204	469	34	64	91	107	765
	7.3%	7.2%	7.9%	8.0%	5.9%	6.6%	6.5%	7.3%
**Alive**	21,020	14,372	4,073	270	589	783	933	6,648
	77.6%	86.5%	68.4%	63.5%	42.9%	72.4%	57.0%	63.5%

Overall, age-standardized five-year survival was significantly higher for type 1 (85.3%, 95%CI 84.4–86.2%) compared to type 2 (51.6%, 95%CI 50.2–53.1%) (Table 3). Within type 2, there was heterogeneity in survival proportions among tumor subtypes: moderate survival for mixed cell high-grade histology at 60.2% (95%CI 55.5–65.0%), followed by high-grade endometrioid at 58.3% (95%CI 56.4–60.2%), and clear cell type at 55.1% (95%CI 48.2–62.0%) in contrast to lower survival for serous EC and carcinosarcoma, at 38.0% (95% CI 34.5–41.6%) and 30.1% (95%CI 26.8–33.5%), respectively. Compared to all populations combined, age-standardized five-year survival was significantly lower among Black women for both type 1 (78.9% vs. 85.3%) and type 2 (40.4% vs 51.6%).

**Table 3:** Age-standardized 5-year EC-specific survival by type, histological subtype, and race/ethnicity. Florida 2005–2016.

Histology		All Combined	Whites	Blacks	Hispanics	Asians
		Survival (95% Confidence Interval)				
**ALL**		70.4% (61.5–71.2)	72.7% (71.7–73.7)	53.4% (50.7–56.2)	72.5% (70.4–74.6)	66.8% (59.2–74.3)
**Type 1**	Low-Grade Endometrioid	85.3% (84.4–86.2)	85.5% (84.4–86.5)	78.9% (75.0–82.7)	86.9% (84.8–89.0)	85.2% (81.3–89.1)
**Type 2**	All Histologies combined	51.6% (50.2–53.1)	53.8% (52.0–55.6)	40.4% (37.0–43.7)	55.0% (51.3–58.8)	†
	High-Grade Endometrioid	58.3% (56.4–60.2)	59.5% (57.2–61.8)	44.0% (38.7–49.3)	63.1% (58.4–67.7)	†
	Clear Cell	55.1% (48.2–62.0)	55.2% (46.6–63.8)	†	†	†
	Mixed High-Grade	60.2% (55.5–65.0)	65.4% (59.9–70.9)	†	†	†
	Carcinosarcoma	30.1% (26.8–33.5)	30.3% (25.8–34.7)	†	30.2% (20.1–40.2)	†
	Serous	38.0% (34.5–41.6)	39.7% (35.0–44.5)	†	†	†

†Not reported due to less than 10 cases in at least one age group.

In Table 4, we report the findings of univariable Fine-and-Gray regression models for all EC cases overall and by EC type. We tested the effect of individual variables on the sub-distribution hazard function for EC mortality, taking into account death from other causes as the competing risk. Significant predictors included age, race/ethnicity, insurance, poverty level, SEER stage, and histological type. A higher risk of cause-specific death was found for Blacks in comparison to Whites (sHR 2.09, 95%CI 1.94–2.26), type 2 EC in relation to type 1 EC (sHR 5.95, 95%CI 5.55–6.37), and carcinosarcoma compared to low-grade endometrioid EC (sHR 12.6, 95%CI 11.4–13.9). Marital status was not a significant predictor of EC-specific mortality and therefore was not included in the multivariable models. Poverty level, which was associated with insurance, was also removed from the multivariable model because insurance status was the more predictive of cumulative incidence of EC mortality between the two variables.

**Table 4:** Univariable Fine-Gray regression models assessing demographic, social, and clinical predictors of risk of EC death in Florida, 2005–2016.

Prognostic Factors/Category	All cases (4,100 EC and 1,969 other cause deaths, 21,020 alive)		Type 1 EC cases (1,043 EC and 1,204 other cause deaths, 14,372 alive)		Type 2 EC cases (3,057 EC and 765 other cause deaths, 6,648 alive)	
	sHR (95%CI)	P	sHR (95%CI)	P	sHR (95%CI)	P
**Age**						
14–44	1 (Reference)		1 (Reference)		1 (Reference)	
45–54	1.07 (0.86, 1.33)	0.569	0.94 (0.64, 1.39)	0.768	0.97 (0.74, 1.27)	0.813
55–64	1.88 (1.54, 2.28)	< .0001	1.70 (1.22, 2.38)	0.002	1.54 (1.21, 1.96)	0.0005
65–74	2.65 (2.18, 3.21)	< .0001	2.27 (1.62, 3.18)	< .0001	1.88 (1.48, 2.39)	< .0001
75+	4.19 (3.45, 5.09)	< .0001	4.07 (2.90, 5.71)	< .0001	2.58 (2.03, 3.29)	< .0001
**Race/Ethnicity**						
Whites	1 (Reference)		1 (Reference)		1 (Reference)	
Blacks	2.09 (1.94, 2.26)	< .0001	1.27 (1.03, 1.55)	0.022	1.59 (1.46, 1.73)	< .0001
Hispanics	0.89 (0.82, 0.97)	0.012	0.77 (0.65, 0.92)	0.004	0.88 (0.80, 0.98)	0.017
Asians	0.93 (0.69, 1.25)	0.636	0.23 (0.07, 0.71)	0.011	1.05 (0.77, 1.42)	0.777
**Insurance**						
Private	1 (Reference)		1 (Reference)		1 (Reference)	
Medicare	1.77 (1.65, 1.90)	< .0001	2.01 (1.74, 2.31)	< .0001	1.41 (1.30, 1.52)	< .0001
Medicaid	2.09 (1.88, 2.32)	< .0001	2.34 (1.90, 2.89)	< .0001	1.63 (1.45, 1.84)	< .0001
No Insurance	1.53 (1.33, 1.76)	< .0001	1.88 (1.44, 2.46)	< .0001	1.18 (1.00, 1.40)	0.048
Unknown	1.47 (1.30, 1.67)	< .0001	1.70 (1.34, 2.16)	< .0001	1.30 (1.12, 1.51)	0.0004
**Census Tract Poverty**						
0%—<5% poverty	1 (Reference)		1 (Reference)		1 (Reference)	
5%—<10% poverty	0.97 (0.88, 1.08)	0.618	1.03 (0.84, 1.27)	0.782	1.01 (0.89, 1.14)	0.894
10%—<20% poverty	1.10 (1.00, 1.22)	0.059	1.16 (0.95, 1.42)	0.135	1.09 (0.98, 1.23)	0.124
20% - 100% poverty	1.30 (1.17, 1.44)	< .0001	1.24 (1.00, 1.54)	0.051	1.20 (1.06, 1.35)	0.003
Unknown or not applicable	0.90 (0.66, 1.24)	0.531	1.00 (0.55, 1.81)	0.994	0.91 (0.62, 1.33)	0.617
**SEER stage**						
Localized	1 (Reference)		1 (Reference)		1 (Reference)	
Regional	4.88 (4.51, 5.28)	< .0001	3.99 (3.48, 4.59)	< .0001	3.37 (3.06, 3.71)	< .0001
Distant	23.17 (21.25, 25.27	< .0001	26.97 (22.17, 32.81	< .0001	10.92 (9.85, 12.10)	< .0001
Unknown	5.90 (5.18, 6.72)	< .0001	6.47 (5.09, 8.22)	< .0001	3.40 (2.91, 3.98)	< .0001
**Type/Histology**						
Type 1, Low-Grade Endometrioid	1 (Reference)		NA		NA	
Type 2, All-combined	5.95 (5.55, 6.37)	< .0001				
**(Detailed Subtype)**						
High-Grade Endometrioid	4.59 (4.23, 4.97)	< .0001	NA		1 (Reference)	0.358
Clear Cell	5.72 (4.73, 6.92)	< .0001			1.24 (1.03, 1.50)	0.022
Carcinosarcoma	12.60 (11.41, 13.92	< .0001			2.72 (2.47, 2.98)	< .0001
Mixed high-grade	4.19 (3.65, 4.82)	< .0001			0.91 (0.79, 1.04)	0.160
Serous	8.22 (7.44, 9.07)	< .0001			1.76 (1.60, 1.93)	< .0001

sHR: subdistribution hazard ratio. HR: hazard ratio. 95%CI: 95% confidence interval. P: two-sided p-value. NA: not applicable.

Table 5 shows the estimated sub-distribution hazard ratios (sHRs) for EC mortality for all EC cases, from multivariable models. We tested interactions with EC type and they were all statistically significant (p = 0.048 for age×type; p = 0.040 for race/ethnicity×type; p = 0.023 for insurance×type; p < .0001 for stage×type). Therefore, we also fit multivariable models by type 1 and type 2 EC in Table 5. In the model including all EC cases, with adjustment for age, race/ethnicity, stage, histology/grade, insurance status, and year of diagnosis, those with type 2 EC had a nearly 3 times higher risk of death from EC compared to those with type 1 EC (sHR 2.98, 95%CI 2.76–3.22, p < .0001). Blacks had a 21% higher risk of EC-specific death (sHR 1.21, 95%CI 1.10–1.33, p < .0001) while Hispanics had a 20% lower risk (sHR 0.80, 95% CI 0.72–0.89, p < .0001) compared to Whites. Those with carcinosarcoma had an approximately 5-times greater risk of cause-specific death compared to those with low-grade endometrioid type (sHR 5.03, 95%CI 4.48–5.66, p < .0001). Among those with type 1, Asian and Hispanic women demonstrated a significantly lower risk of EC-specific death (sHR 0.20, 95%CI: 0.06–0.65, p = 0.007; sHR 0.75, 95% CI 0.62–0.90, p = 0.002, respectively) than White women; however, there was no significant difference between Black and White women (sHR = 1.18, p = 0.147). Meanwhile, for type 2, Blacks showed a significantly higher risk of death from EC in comparison to Whites (sHR: 1.23, 95%CI 1.12–1.36, p < .0001) while Hispanics had a 17% lower risk of death (HR: 0.83, 95% CI:0.74–0.94, p = 0.002), and there was no significant difference for Asians. By histological subtypes, women with carcinosarcoma had the greatest risk of EC death in comparison to the reference category of high-grade endometrioid EC (sHR: 1.89, 95%CI 1.70–2.10, p < .0001), followed by those with serous carcinoma (sHR 1.15, 95%CI 1.04–1.27).

**Table 5:** Multivariable Fine-Gray regression models assessing demographic, social, and clinical predictors of risk of EC death in Florida, 2005–2016.

Prognostic Factors/Category	Model 1 All cases (4,100 EC and 1,969 other cause deaths, 21,020 alive)		Model 2 Type 1 EC cases (1,043 EC and 1,204 other cause deaths, 14,372 alive)		Model 3 Type 2 EC cases (3,057 EC and 765 other cause deaths, 6,648 alive)	
	sHR (95%CI)	P	sHR (95%CI)	P	sHR (95%CI)	P
**Age**						
14–44	1 (Reference)		1 (Reference)		1 (Reference)	
45–54	0.99 (0.80, 1.24)	0.986	0.99 (0.67, 1.46)	0.954	0.98 (0.75, 1.29)	0.983
55–64	1.59 (1.30, 1.93)	< .0001	1.78 (1.27, 2.50)	0.001	1.47 (1.15, 1.87)	0.002
65–74	1.93 (1.58, 2.35)	< .0001	2.41 (1.70, 3.42)	< .0001	1.72 (1.35, 2.20)	< .0001
75+	2.64 (2.15, 3.24)	< .0001	3.78 (2.63, 5.42)	< .0001	2.27 (1.77, 2.92)	< .0001
**Race/Ethnicity**						
Whites	1 (Reference)		1 (Reference)		1 (Reference)	
Blacks	1.21 (1.10, 1.33)	< .0001	1.18 (0.94, 1.48)	0.147	1.23 (1.12, 1.36)	< .0001
Hispanics	0.80 (0.72, 0.89)	< .0001	0.75 (0.62, 0.90)	0.002	0.83 (0.74, 0.94)	0.002
Asians	0.81 (0.59, 1.10)	0.125	0.20 (0.06, 0.65)	0.007	1.00 (0.72, 1.38)	0.976
**Insurance**						
Private	1 (Reference)		1 (Reference)		1 (Reference)	
Medicare	1.13 (1.03, 1.23)	0.006	1.20 (1.01, 1.43)	0.035	1.08 (0.98, 1.20)	0.105
Medicaid	1.36 (1.21, 1.54)	< .0001	1.68 (1.33, 2.12)	< .0001	1.24 (1.08, 1.42)	0.002
No Insurance	1.24 (1.05, 1.45)	0.006	1.75 (1.31, 2.35)	0.0002	1.07 (0.89, 1.29)	0.444
Unknown	1.33 (1.16, 1.51)	< .0001	1.55 (1.23, 1.96)	0.0002	1.25 (1.07, 1.45)	0.005
**SEER stage**						
Localized	1 (Reference)		1 (Reference)		1 (Reference)	
Regional	3.54 (3.26, 3.84)	< .0001	3.96 (3.44, 4.55)	< .0001	3.15 (2.86, 3.48)	< .0001
Distant	12.04 (10.89, 13.31)	< .0001	24.37 (19.85, 29.91)	< .0001	9.78 (8.78, 10.90)	< .0001
Unknown	3.74 (3.26, 4.30)	< .0001	5.62 (4.42, 7.13)	< .0001	3.07 (2.61, 3.60)	< .0001
**Type/Histology**						
Type 1, Low-Grade Endometrioid	1 (Reference)		NA		NA	
Type 2, All-combined	2.98 (2.76, 3.22)	< .0001				
**(Detailed Subtype)**						
High-Grade Endometrioid	2.70 (2.48, 2.94)	< .0001	NA		1 (Reference)	
Clear Cell	3.00 (2.43, 3.70)	< .0001			1.10 (0.90, 1.34)	0.352
Carcinosarcoma	5.03 (4.48, 5.66)	< .0001			1.89 (1.70, 2.10)	< .0001
Mixed high-grade	2.53 (2.18, 2.93)	< .0001			0.92 (0.80, 1.05)	0.216
Serous	3.07 (2.74, 3.44)	< .0001			1.15 (1.04, 1.27)	0.007

sHR: subdistribution hazard ratio. HR: hazard ratio. 95%CI: 95% confidence interval. P: two-sided p-value. NA: not applicable.

Year of diagnosis was included in all models as a categorical variable.

**Fig 1:**
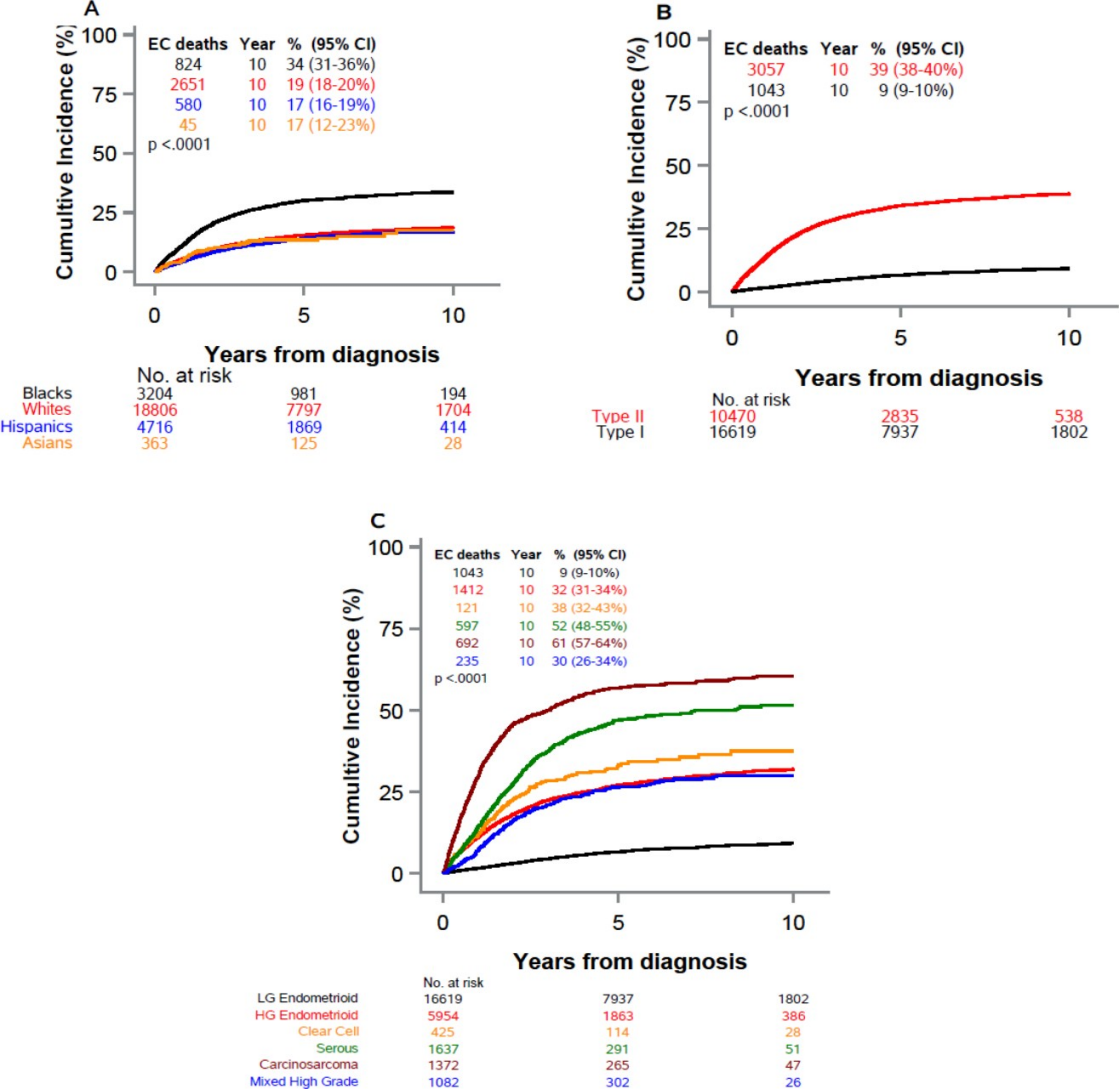
Cumulative incidence of EC mortality, with death from other causes as a competing risk, among women with endometrial cancer (Florida 2005–2016); by race/ethnicity (A), by EC type (B), and for specific type 2 EC by histological subtype (C). Legend: p:-value from Gray’s test comparing cumulative incidence curves. Censored observations not shown. Maximum follow-up truncates at 10 years.

EC-specific cumulative mortality over time varied significantly by race/ethnicity, with Black women experiencing a higher mortality than all other racial-ethnic groups (Fig 1A). Fig 1B presents the cause-specific mortality by EC type. Those with type 2 experienced the bulk of the EC mortality burden. Mortality was low for those with type 1, with the cumulative incidence of EC death reaching only 10% at 10 years. Lastly, Fig 1C depicts the cumulative incidence of EC death by type 2 EC histological subtype. For the carcinosarcoma subtype, the cumulative incidence of EC death reached nearly 60% at 10 years.

## Discussion

Our study revealed complex survival differences according to both histological type and race/ethnicity among women diagnosed with EC in Florida. Type 2 EC is the main driver of EC mortality: while accounting for only 40% of total EC cases overall, it carries significantly worse survival than type 1 and is responsible for 75% of all EC-specific mortality (every 3 in 4 deaths). Moreover, type 2 EC is a very heterogeneous group in terms of survival outcomes. Five-year survival varied two-fold between the most and least favorable subtypes, mixed high-grade and carcinosarcoma, respectively. By race/ethnicity, Black women showed unfavorable survival outcomes in relation to all other populations. These two characteristics, histology and race/ethnicity are not independent. Among Blacks, 58% of all ECs were type 2, which was a significantly higher proportion than in all other populations in which these accounted for a minority of cases. In addition, within type 2 EC, Black women also had the highest proportions of the two histological categories with the lowest survival: carcinosarcoma and serous EC.

Despite this propensity of Black women to have worse types of EC, their significant disadvantage for 5-year survival among all ECs combined persisted after stratification by type 1 and type 2 EC. For type 1, the disadvantage is intriguing given that the overall mortality for this disease is low, and the majority of deaths are due to other causes rather than EC itself. In fact, in the multivariable model and contrary to the univariable model this disparity was no longer evident, suggesting that the Black disadvantage for low-grade endometrioid tumors (type 1 EC) is primarily impacted by a higher stage of disease and/or worse socio-economic factors compared to other groups. However, for type 2, the disparity between Blacks and Whites persisted in the multivariable model, thus highlighting a disadvantage that is not explained by stage, insurance type, or histological subtype within type 2. In summary, the overall racial EC survival disparity is due to both more unfavorable types of EC disproportionately affecting Black women, and also factors that are independent of the commonly assessed predictor variables: histology, stage, grade, and insurance status.

These disparities and uneven distribution of major known clinical prognostic factors of survival (e.g. later stage and higher grade of disease) among Blacks are not unique to Florida and have been described in other studies [18–20, 27, 36, 37]. However, the underlying reasons for these differences remain elusive. Various areas for future research in EC have been suggested in order to clarify this race-specific survival disadvantage. These include the study of differences in access to healthcare, and research on mistrust and attitudes toward the healthcare system among minority older women [18, 27]. In addition, different levels of health education between populations are a possibility. In particular, there may be differences in the recognition of the importance of postmenopausal vaginal bleeding by racial/ethnic group, a key sign of possible EC which warrants medical investigation and can affect timely treatment [38].

It is fundamental to explain the excessive proportions (relative terms) and incidence (absolute terms) of the less favorable types and subtypes (e.g. type 2 EC, particularly carcinosarcoma and serous EC) between Blacks and other races, which suggest biological, epigenetic, and genomic differences [18]. This preponderance of more aggressive EC types seems to be a common feature among Black women of different origins since high EC mortality patterns (all histologies combined) have been noted across all populations of African descent in the U.S.: U.S.-born African Americans, Caribbean-born Blacks and African-born Blacks [39]. Yet, little is known regarding histology-specific risk factors for these more aggressive type 2 subtypes [19], and to what extent these may, in fact, constitute different disease entities. Currently, all type 2 subtypes follow relatively similar treatment regimens [40], despite remarkably different outcomes as shown in this study. This lack of differentiated treatment is further aggravating disparities for Blacks [41] given that their EC subtype distribution lies toward the more unfavorable end of the spectrum, with a higher proportion of both carcinosarcoma and serous EC.

While previous research has described the potential complex molecular differences between EC among Black and White women [42–44], future studies should also address the joint role of genetics, admixture, and cultural factors, including geographic region of origin, specifically among Black populations [45]. In this instance, the Florida population constitutes a unique ground for the study of specific minority populations and immigrants: Caribbean-origin populations account for as many as 30% of EC deaths among Blacks [22–24]. The detailed study of Black (e.g. Haitians) and Hispanic populations that have a higher degree of Black admixture (e.g. Dominicans and Puerto Ricans) [46] in this state may provide information not available elsewhere. In a hospital-based study conducted in Florida, Schlumbrecht *et al*. reported that EC high-grade types may be more common among Afro-Caribbeans than U.S.-born African Americans [47], an important finding for understanding the epidemiology of EC that should be confirmed in wider population-based studies.

There are several strengths to be noted in this study which include the population-based nature of the data. Our data includes all cases of EC in Florida (i.e., cases with all different types of insurance, as well as cases who sought care in all types of health facilities, etc), which is one of the fastest growing and most diverse states in the US. This characteristic exempts our study from different forms of selection bias that are possible in hospital-based studies. However, our study is not without limitations. First, we did not assess the different modalities of treatment and/or type of chemotherapy received; unfortunately, these are not always complete in registry data. Second, the lower survival among Black women could be attributed to existing disparities in access to care and treatment decisions [48–50] that we could not assess. However, even after controlling for these factors, a recent study found a persisting disadvantage for Black women [18]. Third, the Florida registry conducts passive follow-up, which tends to overestimate survival for majority foreign-born populations such as Hispanics and Asians [51, 52]. In our study, survival outcomes for these racial/ethnic groups varied by EC type. For instance, among Hispanics, an apparent survival advantage for both types 1 and 2 was observed in relation to Whites. For Asians, after adjustment, survival was higher in comparison to Whites, but only for type 1 EC. Therefore, the Hispanic (and Asian) survival advantages in this study should be interpreted with caution and certainly warrant further study. Lastly, we did not have body-mass index or comorbidity data, which are both known to be higher or more prevalent among Black populations [53]. However, this limitation was largely reduced since we studied cause-specific rather than all-cause survival.

In conclusion, EC is a heterogeneous disease with substantial variation in survival according to major histological type. The EC mortality burden is not equally distributed across racial/ethnic groups. Not only do Black women experience higher proportions of histology/grade types associated with worse survival but also within each histological category and stage at diagnosis, their survival is lower, suggesting an intricate context of disparities for EC. Racial/ethnic survival disparities in EC are driven by the less common type 2, and therefore future population-based analyses on EC disparities should take this histological heterogeneity across populations into consideration. In particular, endometrioid categories that historically have been grouped together should be disaggregated given the survival difference between low-grade (85% after 5 years) and high-grade endometrioid (58% survival after 5 years) ECs. In light of the remarkable disparity affecting Black women as well as the increasing trends in EC incidence and mortality, especially among minority populations [54], more research on molecular determinants, risk factors, and survival outcomes specifically for type 1 and in particular type 2 subtypes is imperative.
